# 684. Characteristics of Patients with Initial *Clostridioides difficile* Infection (CDI) That Are Associated With Increased Risk of Multiple CDI Recurrences

**DOI:** 10.1093/ofid/ofad500.746

**Published:** 2023-11-27

**Authors:** Alice Guh, Rongxia Li, Lauren C Korhonen, Lisa Gail Winston, Erin Parker, Christopher A Czaja, Helen Johnston, Elizabeth Basiliere, James Meek, Danyel M Olson, Scott Fridkin, Lucy E Wilson, Rebecca Perlmutter, Stacy Holzbauer, Paige D’Heilly, Erin Phipps, Kristina Flores, Ghinwa Dumyati, Rebecca Pierce, Valerie Ocampo, Christopher Wilson, Jasmine Watkins, Dale Gerding, L Clifford McDonald

**Affiliations:** CDC, Atlanta, Georgia; CDC, Atlanta, Georgia; CDC, Atlanta, Georgia; UCSF, San Francisco, California; California Emerging Infections Program, Oakland, California; Colorado Department of Public Health and Environment, Denver, Colorado; Colorado Department of Public Health and Environment, Denver, Colorado; Colorado Department of Public Health and Environment, Denver, Colorado; Connecticut Emerging Infections Program, Yale School of Public Health, New Haven, Connecticut; Connecticut Emerging Infections Program, Yale School of Public Health, New Haven, Connecticut; Georgia Emerging Infections Program, Decatur, GA; Emory University School of Medicine, Atlanta, GA, Atlanta, Georgia; University of Maryland Baltimore County, Baltimore, Maryland; Maryland Department of Health, Baltimore, Maryland; Minnesota Department of Health, St Paul, Minnesota; Minnesota Department of Health, St Paul, Minnesota; University of New Mexico, New Mexico Emerging Infections Program, Albuquerque, NewMexico; University of New Mexico, New Mexico Emerging Infections Program, Albuquerque, NewMexico; New York Emerging Infections Program and University of Rochester Medical Center, Rochester, New York; Oregon Health Authority, Portlant, Oregon; Oregon Health Authority, Portlant, Oregon; Tennessee Department of Health, Nashville, Tennessee; Tennessee Department of Health, Nashville, Tennessee; Edward Hines, Jr. Veterans Affairs Hospital, Hines, Illinois; CDC, Atlanta, Georgia

## Abstract

**Background:**

Recent advancements in treating *Clostridioides difficile* infection (CDI) include therapeutics to prevent further recurrence in patients with recurrent CDI (rCDI). However, little is known about which patients are at increased risk for multiple recurrences (≥ 2 rCDI). We sought to identify predictors of multiple rCDI (mrCDI) in adults at the time of presentation with initial CDI (iCDI).

**Methods:**

The Centers for Disease Control and Prevention’s Emerging Infections Program (EIP) conducts population-based CDI surveillance in 10 U.S. sites. We defined iCDI as a positive *C. difficile* test during January 2018–August 2019 in a person aged ≥ 18 years with no prior positive test reported to EIP. rCDI was defined as a positive test ≥ 14 days from the previous positive test within 180 days after iCDI. All patients with community-onset iCDI and a random sample of patients with healthcare-facility onset iCDI (i.e., hospital-onset, long-term care facility onset) had full chart reviews. Multiple imputation was performed on missing race/ethnicity. Candidate variables determined *a priori* to be potentially associated with mrCDI were entered into an initial multivariable logistic regression model. Patients without mrCDI who died within 180 days of their iCDI were excluded from the model. Candidate variables with a p-value < 0.1 in the initial model were included in the final model.

**Results:**

Of 18,829 patients with iCDI, 882 (4.7%) had mrCDI, ranging from 2 to 5 rCDI per patient in the 180 days following iCDI. Median time between each rCDI was 43 days (interquartile range: 27–65 days). Full charts were reviewed for 435 iCDI patients with mrCDI and 7474 iCDI patients without mrCDI. Characteristics of patients with and without mrCDI are shown in Table 1. In multivariable analysis, age ≥ 65 years, recent hospitalization, chronic hemodialysis, and recent nitrofurantoin use were significantly associated with mrCDI (Table 2).

**Figure:**
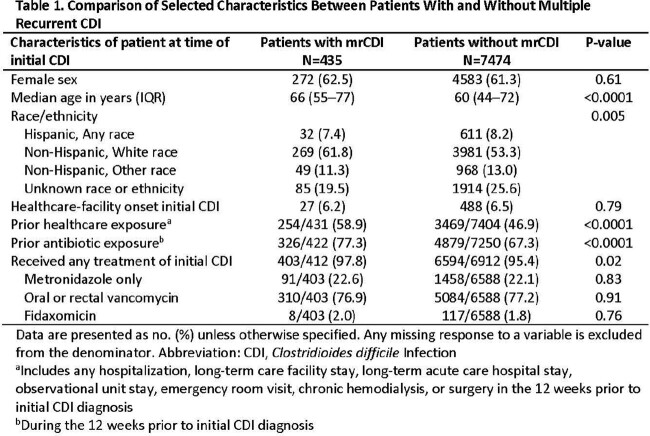

**Figure:**
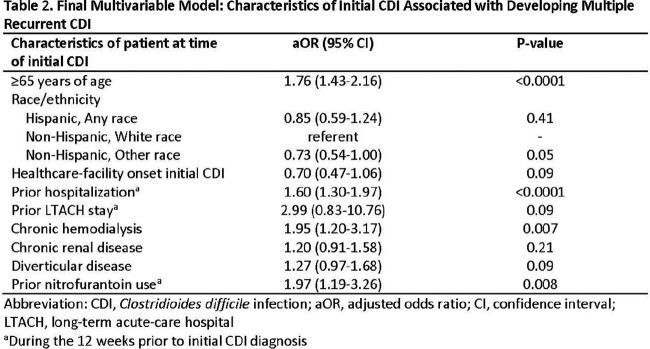

**Conclusion:**

Patients with iCDI who are older, on hemodialysis, or had recent hospitalization or nitrofurantoin use may be at increased risk of mrCDI and may benefit from early use of adjunctive therapy to prevent mrCDI. If confirmed, these findings would aid in clinical decision making.

**Disclosures:**

**Ghinwa Dumyati, MD**, Pfizer: Grant/Research Support

